# Evidence of Coat Color Variation Sheds New Light on Ancient Canids

**DOI:** 10.1371/journal.pone.0075110

**Published:** 2013-10-02

**Authors:** Morgane Ollivier, Anne Tresset, Christophe Hitte, Coraline Petit, Sandrine Hughes, Benjamin Gillet, Marilyne Duffraisse, Maud Pionnier-Capitan, Laetitia Lagoutte, Rose-Marie Arbogast, Adrian Balasescu, Adina Boroneant, Marjan Mashkour, Jean-Denis Vigne, Catherine Hänni

**Affiliations:** 1 Paléogénomique et Evolution Moléculaire, Institut de Génomique Fonctionnelle de Lyon, Université de Lyon, Université Lyon 1, CNRS UMR5242, Ecole Normale Supérieure de Lyon, Lyon, Cedex 07, France; 2 CNRS/MNHN-UMR 7209 Archéozoologie, Archéobotanique : Sociétés, Pratiques et Environnements, Département Ecologie et Gestion de la Biodiversité, Paris, France; 3 Institut de Génétique et Développement de Rennes, CNRS-UMR6290, Université de Rennes1, Rennes, France; 4 CNRS/ENS de Lyon, French National Platform of Paleogenetics, PALGENE, Ecole Normale Supérieure de Lyon, Lyon, Cedex 07, France; 5 CNRS-UMR 7044 Maison Interuniversitaire des Sciences de l′Homme-Alsace, Strasbourg, France; 6 The National Museum of Romanian History, Centre National de Recherches Pluridisciplinaires, Bucharest, Romania; 7 Institute of Archaeology “Vasile Pârvan” of, the Romanian Academy, Bucharest, Romania; BiK-F Biodiversity and Climate Research Center, Germany

## Abstract

We have used a paleogenetics approach to investigate the genetic landscape of coat color variation in ancient Eurasian dog and wolf populations. We amplified DNA fragments of two genes controlling coat color, *Mc1r* (Melanocortin 1 Receptor) and *CBD103* (canine-β-defensin), in respectively 15 and 19 ancient canids (dogs and wolf morphotypes) from 14 different archeological sites, throughout Asia and Europe spanning from ca. 12 000 B.P. (end of Upper Palaeolithic) to ca. 4000 B.P. (Bronze Age). We provide evidence of a new variant (R301C) of the Melanocortin 1 receptor (*Mc1r)* and highlight the presence of the beta-defensin melanistic mutation (*CDB103*-K locus) on ancient DNA from dog-and wolf-morphotype specimens. We show that the dominant K^B^ allele (*CBD103)*, which causes melanism, and R301C (*Mc1r)*, the variant that may cause light hair color, are present as early as the beginning of the Holocene, over 10 000 years ago. These results underline the genetic diversity of prehistoric dogs. This diversity may have partly stemmed not only from the wolf gene pool captured by domestication but also from mutations very likely linked to the relaxation of natural selection pressure occurring in-line with this process.

## Introduction

The dog (*Canis lupus familiaris*), the first domesticate, stems from the wolf (*Canis lupus*). It was domesticated by hunter-gatherer communities during the Upper Paleolithic certainly earlier than 15 000 cal. BP (Before Present) [1]–[5]. The number of domestication events, and their origins, has long been debated. Some molecular data supported the hypothesis of a unique domestication event in South-East Asia [2], [6], [7]. However, other molecular data have revealed that most of the modern dog breeds derive from a restricted gene pool and that significant genetic diversity was probably lost during the process of selecting for modern breeds [8]–[11]. Archaeological data has long pleaded for a multi-centered origin of domestication. Animals displaying traits considered as domestic (small size, tooth crowding, skull and mandible shortening, brain case volume reduction) have been found in close association with humans in the Upper Palaeolithic and Epi-Palaeolithic (prior to 12 000–11 000 cal. BP) of different Eurasian regions. At this time, the regions of the Near East (Tell Mureybet, Ein Mallaha and Hayonim in the Levant [12]; Pelagawra cave in the northern Zagros [13], Siberia (Eliseevichi I, Upper Palaeolithic; [3]) and western Europe (Montespan, Pont d’Ambon, Erralia, Le Closeau and several other Upper Palaeolithic sites; see review in [4]) had no cultural ties. Thus, several populations of wolves may have been at the origin of these domestication events [14]–[18] and early dogs were probably characterized by a significant genetic variability. Zooarchaeological data provides evidence of substantial phenotypic variations [4], [16].

Experiments have shown [19] that one of the first and rapid effects of animal domestication is the loss of the wild coat color. However, nothing is known of the coat phenotypes of ancient wolves, which were domesticated. The earliest variants resulting from domestication and human selection, ultimately leading to modern dog coat colors, are also unknown. The complexity of the interplay between variation stemming from lift of natural pressures and human-induced selections was reconstructed in the case of pig [20]. The identification of genes encoding coat color, together with the analysis of DNA sequence variants, in ancient Eurasian dogs provides information on their phenotypic traits and on the diversity of this trait in ancient populations. Paleogenetics allow to finally shed light on the genetic landscape related to coat color variation in ancient dog populations.

Genomic data available for present-day dogs (eg Boxer reference annotated sequence [21], Single Nucleotide Polymorphims (SNPs), Copy Number Variations (CNVs)) provide access to information on specific genes and the underlying alleles controlling coat color variation.

Our study focuses on two genes implicated in coat color variation and which regulate the production of red/yellow (phaeomelanin) versus brown/black (eumelanin) pigment [22], [23]: *Mc1r* (Melanocortin 1 Receptor) and *CBD103* (canine-β-defensin) (Table S1). *Mc1r* encodes for a seven-transmembrane receptor expressed in melanocytes that activates the production of eumelanin. *CBD103* in its mutated form – codon deletion named ΔG23, allele K^B^ [23]-is a ligand of *Mc1r* and is implicated in pigment-type switching that promotes production of eumelanin. Three major coat color phenotypes can be distinguished based on the three following criteria: *(1)* agouti coat color (wild type) is observed when both genes are in the wild type form (alleles E and K^y^ respectively for *Mc1r* and *CBD103*), *(2)* recessive yellow coat color is observed when *Mc1r* is mutated (allele e, SNP R306ter [22]) and *(3)* dominant black coat color when *Mc1r* presents wild type form and *CBD103* is mutated (allele K^B^, K locus).

To determine the coat color pattern, we performed ancient DNA (aDNA) analysis from tooth and bones remains of 68 different canids (66 animals displaying a dog-morphotype and 2 animals displaying a wolf-morphotype) from Upper Palaeolithic (ca. 12 000 cal. BP) to Bronze Age (ca. 4000 cal. BP), from 28 different archeological sites, throughout Asia and Europe (Table S2). Mitochondrial DNA of these samples had been successfully amplified by PCR as part of a previous project ( [12]; data not shown), indicating that endogenous DNA was preserved in these specimens.

## Results and Discussion

For each sample, we amplified a 79 bp fragment of *Mc1r* and a 76 bp fragment of *CBD103* including respectively the R306ter (*Mc1r*) and ΔG23 (*CBD103*) variants, by designing specific dog primers (see Materials and Methods section). While working with aDNA is always a challenge, adequate tools, controls and analyses are routinely practiced in our lab [24]–[27] and whenever possible, three positive independent PCR amplifications were analysed for each sample and each gene (Table S3). The amplicons were sequenced by high-throughput sequencing technology (454, GS Junior) with an average of about 422 reads by amplicon.

Eventually, reproducible amplifications and sequencing products were obtained for *Mc1r* and *CBD103* fragments in respectively 15 and 19 ancient canids (dog- and wolf-morphotypes). Sequences were deposited in the GenBank Data Library under accession numbers JQ971988 to JG972027. Eleven of these ancient canid samples yielded both *Mc1r* and *CBD103* fragments (Table 1, Table S2).

**Table 1:** Allelic states of 21 ancient individuals with dog-morphotype and 2 ancient individuals with wolf-morphotype at the R301C, R306ter and K locus.

Sample reference	Country	Archeological site(ID number)	Dating (cal BP)	MC1R New mutation R301C	Coat color: Genotype/Inferred phenotype*		
					MC1R R306ter	CBD103 ΔG23	Hypothetical coat color
CH734	France	Bury (14)	[4451–4247]^1^	C/C	C/C	−/−	Black
CH735				C/C	C/C	GGG/GGG	Yellow (wild type)
CH716	Ukraine	Luka Vrubiletskaia (13)	[4950–4650]^3^	ND	ND	GGG/GGG	?
CH717	Moldavia	Soloncheny (12)	[4950–4650]^3^	ND	ND	GGG/GGG	?
*CH1075*	Turkmenistan	Ulug Depe (11)	[5450–3950]^2^	C/C	C/C	GGG/−	Black
CH1076				ND	ND	GGG/GGG	?
CH756	France	Saint Paul Trois Chateaux (10)	[5900–5600]^3^	ND	ND	GGG/GGG	?
CH1047	Switzerland	Twann (9)	[6150–5400]^3^	C/C	C/C	ND	?
CH773	Romania	Borduşani (8)	[6798–5900]^1^	ND	ND	GGG/GGG	?
CH768		Hârşova (7)	[6500–5900]^3^	ND	ND	GGG/GGG	?
CH770				**T/T**	C/C	ND	?
CH771				C/**T**	C/C	−/−	Black
CH1042	Germany	Herxheim (6)	[7030–6960]^3^	C/C	C/C	GGG/GGG	Yellow (wild type)
CH767	Romania	Isaccea (5)	[7440–7318]^2^	**T**/**T**	C/C	GGG/−	Black
CH708	Russia	Pad’Kalashnikova (4)	[10233–9909]^3^	C/**T**	C/C	GGG/GGG	Yellow (wild type)
CH709				C/**T**	C/C	GGG/GGG	Yellow (wild type)
CH710		Ust’belaya (3)	[10233–9909]^2^	ND	ND	GGG/GGG	?
CH711				C/**T**	C/C	GGG/GGG	Yellow (wild type)
CH712				**T**/**T**	C/C	GGG/GGG	Yellow (wild type)
CH1119	Romania	Icoana (2)	[10876–7960]^2^	ND	ND	GGG/GGG	?
CH1120				**T**/**T**	C/C	−/−	Black
CH1122				**T**/**T**	C/C	ND	?
*CH1244*	Russia	Torgashinskaya cave (1)	[15000–12000]^3^	C/C	C/C	ND	?

* Hypothetical coat color can be deduced considering allelic state of R306ter and K locus. *In italic*: individuals with a wolf-morphotype (CH1075 and CH1244). ND: not determined, no positive amplification; ?: Hypothetical coat color could not be deduced;−/−: *CBD103*: ΔG23 mutation.

1 Date obtained directly on dog bones (cal BP);

2 Date derived from other remains (BP);

3 Chronological periods derived from cultural attributions.

### A New *Mc1r* Gene Variant in Ancient Dogs with Potential Phenotypic Effect

We did not detect the R306ter allele in any of our samples. However, analysis of the 79 bp *Mc1r* fragment revealed a new polymorphism in each of the 9 dog samples coming from 5 archeological sites in South-Eastern Europe and Asia (Siberia) (Table 1, Upper Paleolithic to Mesolithic and Neolithic periods). This mutation (C->T) is non-synonymous and encodes an Arginine ->Cysteine substitution at the position 301 of the amino acid chain (R301C). The R301C substitution has not been yet described in canids. The R301C substitution was present in amplification products of these 9 individuals at frequencies ranging from 25 to 100% indicating that five individuals were homozygous and four were heterozygous at this locus (Table 1, Table S3, Table S4).

We estimated the percentage of total degradation and percentage of C to T substitution along *Mc1r* sequences. We estimated at 0.82% and 0.67% respectively the number of degraded sites along *Mc1r* sequences for the group of 9 samples bearing the R301C mutation and the group of 6 samples without the R301C mutation. The numbers of C->T substitutions in these groups are 1.48% and 0.72% respectively. There was no significant difference between the two groups of samples (T-test, p value = 0.48>>0.05). Also, the R301C substitution was not associated with a C-to-T degradation [28] at other nucleotidic sites and the substitution was found at a similar frequency for independent amplification products of a given sample (Table S4).

**Figure 1:**
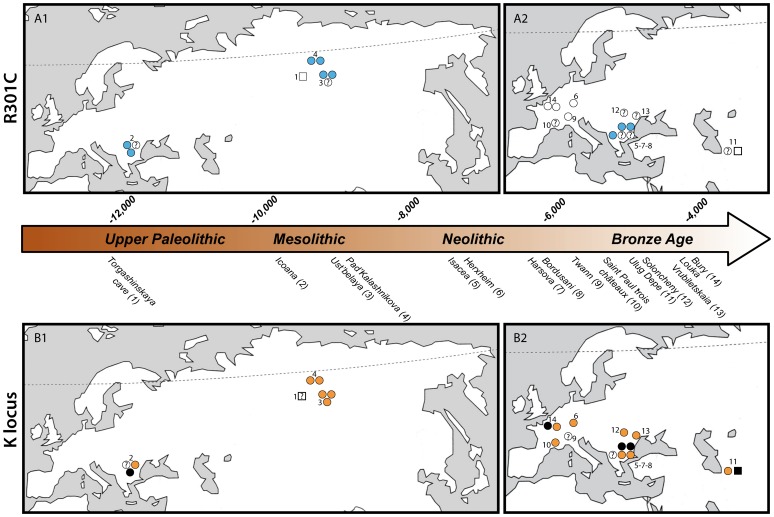
Distribution of the R301C mutation (A1, A2) and of the K^B^ allele (B1, B2), before (A1, B1) and after (A2, B2) the neolithisation: presence of R301C mutation (blue), absence of R301C mutation (white), presence of K^B^ allele (black), no presence of K^B^ allele (orange), undetermined (question mark). Squares and dots refer respectively to individuals with wolf-and dog-morphotype.

All dog-morphotype samples (n = 9) coming from Asia (Ust’belaya (site 3), Pad’Kalashnikova (site 4; Figure 1)) and South-Eastern Europe (Icoana (site 2), Isaccea (site 5), Hârşova (site 6; Figure 1)) for which *Mc1r* was amplified, present the R301C substitution (Table 1). Conversely, in this macro-region, the two amplified sequences from samples from animals displaying a wolf-morphotype did not bear this mutation (at Torgashinskaya cave ca. 15000 to ca. 12000 cal. BP in an Upper Palaeolithic context and at Ulug Depe ca. 5550– ca. 4000 cal. BP in a Bronze Age context). Our results highlight the presence of R301C as early as approximately 11 000 cal. BP in dogs of Asia (Siberia) and South-Eastern Europe (Figure 1).

While it has not been described in canids, we found R301C present in the Genbank database on sequences of present-day dogs belonging to two arctic breeds (Siberian Husky and Alaskan Malamute), whereas it was absent in the Boxer, Saluki and Afghan Hound. To further investigate the presence of this mutation in a larger dataset of present-day wolves and dog breeds we amplified the almost complete *Mc1r* coding sequence, consisting of a single exon, in 6 wolves and 41 dogs from 13 breeds (deposited in the GenBank Data Library under accession numbers JX273571 to JX273640). Six breeds belong to well-supported groups of highly divergent ancient breeds [15] that can be sub-divided in a northern group (Alaskan malamute and Siberian husky), an Asian group (Chow-chow, Akita and Chinese Shar Pei), and a Middle Eastern group (Saluki). We also selected two breeds closely related to ancient breeds (Saluki and Samoyed) and five breeds of modern domestic dogs (Beagle as representative of scent hounds breeds, Chihuahua from the toy breed group, Greyhound from sight hound breed group, Tibetan Dogues and Boxer from Mastiff-like and Molossoid breeds group).

We only retrieved the R301C substitution in Siberian Husky and Alaskan Malamute (Table S5). We never retrieved the R301C mutation in any samples from present-day grey and arctic wolves *(Canis lupus arctos)* or dogs from other breeds but identified the R306ter mutation [15], [29] in several dog breeds (Table S5).

Interestingly, a mutation at the same position ‘301’, namely an amino-acid change from Arginine->Serine was detected in the *Mc1r* sequence of a 43,000-year-old mammoth together with two other polymorphic positions [30]. In addition, this study shows that the product of the tri-allelic (Thr21-Arg67-Arg301) haplotype leads to an almost complete loss in basal activity and a 65% reduction in agonist efficacy to *Mc1r*, sufficient to result in substantially lighter hair color. The effect of the R301C mutation alone on coat color is not well established and we could not affirm that it is functional.

It can be hypothesized that this mutation reflects a relaxation of selection pressures due to domestication. However, the presence of the same polymorphism in another wild mammal species ([30], see above), which never underwent the domestication process, rather supports the case that it is an ancient polymorphism present in wild populations.

From the 13 breeds tested in our study, we show that the R301C mutation is only observed in *Mc1r* sequences of the two arctic breeds sampled. Arctic breeds originated North of the polar circle, ranging from Eurasia to America [18]. The Siberian Husky breed originated in Siberia whereas the Alaskan Malamute breed was selected in Alaska. Arctic breeds are commonly considered as old and appear as basal taxa on phylogenetic trees of modern dog breeds [9], [15]. This is probably due to a lack of recent admixture with other dog breeds facilitated by geographic and cultural isolation [18]. In this context, presence of R301C in the genome of modern arctic breeds reflects an ancient variant either present in ancient populations of wolves or present in early-domesticated dogs in Asia and/or South-Eastern Europe. Thus we hypothesize that a pool of early dogs from South-Eastern Europe and/or Asia may have migrated to the North, crossed the Bering Strait to North America 14 000 to 12 000 years ago before being isolated by the sea level rise which separated the two continents at the beginning of the Holocene. This is in accordance with conclusions by Leonard *et al.* [8] who postulated the dogs from Alaska originated from multiple Old World lineages of dogs that accompanied late Pleistocene humans across the Bering Strait.

We detected the R301C mutation both in Asian and South-Eastern European ancient dog samples (Figure 1) but not in Western Europe. In the East of Eurasia (Eastern Europe and Asia) this distribution could result either from independent mutational events leading to the presence of the R301C allele in the whole area or the mutation could have occurred in one place and have spread all over this macro-region.

The difference in frequency observed in the Western part of Europe, where the mutation has not been detected, suggests either: (i) that R301C was already present at a relatively high proportion in the wolf gene pool captured by domestication in Asia and South-Eastern Europe while it was substantially less frequent in the wolf gene pool captured in Western Europe; or it could mean (ii) that the mutation occurred randomly at least in Asia and South-Eastern Europe where it was selected by humans at an early stage of breeding whereas it was not selected to the same extent in the West.

The fact that the mutation could have relatively limited effects on the phenotype makes the first hypothesis more likely. This pleads for a relative greater genetic diversity of Eurasian prehistoric wolves during the end of the Pleistocene. Further analysis of ancient canid remains on longer sequences from Asia and South-Eastern Europe region will help us decipher which scenario is most likely.

### On the History of Black Coat Color in Canids

Given the allelic state of *Mc1r* R306ter and *CBD103* K loci, we can deduce a hypothetical coat color, inferred from the mutations observed for 11 ancient canids (Table 1) (NB coat color may be regulated by several more genes).

As the K^B^ allele is dominant, we hypothesize that the coat color was likely to be black for four dog-morphotype specimens from the Mesolithic (ca. 11 000–ca. 8 000 cal. BP) to the Bronze Age (ca. 4000 cal. BP) in Europe (from Icoana (site 2), Isaccea (site 5), Hârşova (site 7) and Bury (site 14); Figure 1) and a Bronze Age wolf-morphotype specimen in the Near-East (Ulug Depe (site 11); Figure 1). Six of the samples showed a hypothetic agouti type pattern. It was not possible to infer the coat color of the remaining individuals because aDNA amplification was unsuccessful.

The three-glycine deletion in the second exon of *CBD103* (ΔG23 mutation), encoding the K^B^ dominant allele, was identified in a Mesolithic dog-morphotype specimen from South-Eastern Europe (Icoana) highlighting that this mutation and the subsequent black phenotype have been present in dog for 8 000 years at least. We also retrieve this mutation later on during the Neolithic and the Chalcolithic/Bronze Age, in three dog-morphotype specimens from South-Eastern and Western Europe (Isaccea, Hârşova and Bury) and in the specimen bearing a wolf morphotype at Ulug Depe (Figure 1, Table 1, Table S4).

The K^B^ allele is widely distributed among present-day domestic dogs, including ancient breeds originating in Asia and Africa. In contrast, in present-day wolves, melanism has been reported outside North America only in Italy, where it is associated with molecular and/or morphological evidence of recent hybridization with free-ranging dogs [31]. Anderson *et al.* [32] hypothesized that the melanistic K locus mutation in North American wolves could derive from past hybridization with domestic dogs. They suggested that the K^B^ mutation arose in dogs in Eurasia and was later distributed among wolves by interspecific hybridizations with dogs that accompanied humans across the Bering Strait 14 000 to 12 000 years ago. These observations point to the fact that the melanistic K locus mutation is extremely rare in the wild (excluding hybridized populations) as it is not passed on to further generations when it occurs randomly. Interestingly, the Ulug Depe sample displaying a wolf osteological morphotype associated with a K^B^ allele is heterozygote at the K locus. Due to the dominant inheritance of K^B^, this individual is very likely to have had a black coat (Figure 1). This can suggest either that: (i) this individual is an early product of the domestication process and the presence of a mutated allele at locus K reflects a concurrent relaxation of selection pressures; (ii) this individual is a hybrid from a (black) dog and a wolf, as hybridization between wild and domestic populations is observed [31].

Our results suggest that the K^B^ allele was present in European dogs as early as the Mesolithic 11 000 to 8 000 cal. BP (a remain from an homozygote dog-morphotype specimen has been found in Icoana, Romania, Table 1). To our knowledge there is no earlier evidence of “black” dogs (nor wolves) in Eurasia. Our experimental data obtained from aDNA analysis firmly set *an ante quem* at 11 000–8 000 cal. BP (Icoana calibrated radiocarbon date) for the first occurrence of this mutation in Eurasia. This is congruent with the hypotheses derived by Anderson *et al.* from present-day canid studies [32] estimating, based on coalescent time analysis, that K^B^ had originated in the Old World and is at least 46 886 years old (121 182 years to 12 779 BP). These data would suggest that early black dogs of Eurasia could have migrated since the Upper Palaeolithic to North America across the Bering Strait and be at the origin of the present-day American black wolf populations, via a back-crossing process with local wolves.

## Conclusions

The K^B^ and R301C coat color alleles have been observed from 11 000–8000 cal. BP in different geographical areas with no clear-cut distribution of alleles according to time.

In this study, we provide evidence for the variation of the allelic state of *Mc1r* (by describing a new SNP R301C) and *CDB103* (K^y^ and K^B^) in ancient dog-morphotype specimens from the Mesolithic period (ca. 10 000 cal. BP) especially in Asia and South-Eastern Europe to the Bronze Age (ca. 4 000 cal. BP), in the Middle East and Western Europe. We show the occurrence of both K^B^ (*CDB103*) and R301C (*Mc1r*) alleles in South-Eastern Europe relatively early-on in the history of dog breeding. This is potentially an indicator of the genetic diversity of early-domesticated dogs in this region. Our data, when considered together with observations of modern breeds, support the idea that the mutation R301C (*Mc1r*) occurred very early on in dog evolution history. There is even a possibility that it occurred in pre-domestic wolf populations and was captured and retained by the domestication process in some areas of Asia and South-Eastern Europe. The story may have been different for K^B^ (*CDB103*). Unlike R301C (*Mc1r*), this mutation has dramatic effects on the phenotype (black coat color in homozygote and heterozygote individuals). Modern data regarding its current distribution in wolf populations (it is rare and only occurs in hybridized populations) suggest that there was a strong natural selection against this mutation in strictly wild contexts. Thus its frequency is likely to have risen in connection with the relaxation of natural selection pressures. Our data show that this mutation occurred and was retained early on in the history of dog breeding (11 000 to 8 000 years ago, in South-Eastern European Mesolithic context). Further analyses, on larger samples of ancient Eurasian dogs and wolves from the Paleolithic to Bronze Age would help to define the inheritance of both alleles during the first stages of domestication. Investigating all variants of *Mc1r* and *CBD103* and sequencing other genes (i.e. *Agouti*, Merle etc) are required to better determine the coat color variation of ancient dog and wolf and further reveal their entwined stories.

## Materials and Methods

### Discrimination of Dog and Wolf Morphotype Samples

Dog is not easily distinguishable from its wild (wolf) ancestor at the beginning of the domestication process. For this reason, we prefer to refer to the terms “dog-morphotype” and “wolf-morphotype”, without implying strong assumptions regarding the real status of the analyzed animals. In our study, wolf-morphotypes do not differ significantly from ancient pre-domestication wolf (100 000 to 60 000 years BP; [10]) or from modern wolf specimens. Neither does the identification of the wild form raise major problems. On the other hand, dog-morphotypes differ from this baseline by one or several of the following criteria: significantly smaller overall size, decrease of the brain-case volume, shortening of the snout, tooth crowding [4], [18], [33].

### Dating of Ancient Samples

Samples analyzed in this study have been directly radiocarbon dated when possible. Otherwise they were dated by association with other well directly dated bioarcheological remains (bones, macro-remains) or by association with cultural artefacts. This is specified for each sample in Table 1 and Table S2.

### Ancient DNA

#### Ancient DNA extraction

The external surface of bones was scratched with a sterile scalpel and the cleaned part remaining was reduced to powder with a sterile hammer. The powder (150–300 mg) was then digested for 18 h at 55°C with agitation in 4.7 ml of buffer (0.5 M EDTA – ethylene diamine tetra acetic acid, pH = 8.0), 50 ul of proteinase K (1 mg/mL) and 250 ul of 0.5% N-lauryl-sarcosine [24]. A silica-based method modified from Rohland and Hofreiter [34] was used to retrieve DNA. Mock extractions were performed in order to rule out contamination from reagents. Furthermore, cross-contamination was monitored using samples from other species (sturgeons and sheep) in every single dog DNA extraction session. All the ancient DNA extractions were conducted on the French National Platform of Paleogenetics (PALGENE, CNRS, ENS de Lyon) devoted to ancient DNA analyses with facilities for analyzing ancient DNA.

#### Ancient amplification and sequencing

To avoid contamination, pre-amplification procedures (on PALGENE platform) and post-amplification analyses (in post-PCR molecular lab) were performed in independent and physically distant rooms. We systematically added 3 different controls in all PCR assays: an aerosol control (tube kept open throughout the manipulation [24] to monitor airborne contaminations, a blank extraction and a PCR-mix control to monitor contamination of reagents). Two nuclear fragments were targeted resulting in the amplification of partial sequences of *Mc1r* and *CBD103* genes. A 79 bp fragment of the *Mc1r* gene was amplified using the following primers: Primer F 5′-CTGCAACTCCATCATTGACC-3′ [15] and *Mc1r*-MOa-R 5′-TAGCACTACCTCTTGGAGAG-3′. An 76 bp fragment of *CBD103* was amplified using primers *CBD103*-MOa-F 5′-AAGCTTTCCGGCACGTTCTG-3′ and *CBD103*-MOa-R 5′-GCAATAATAYCTCTGCAGGG-3′.

To amplify DNA extracts, a multiplex PCR was carried out. In a first round the two pairs of primers were mixed in a single tube. The reaction was performed in a 25 µL reaction volume containing 2.5 µL of 10X Taq buffer (Applied Biosystems), 2 mM of MgCl_2_ (Applied Biosystems), 0,025 mg of BSA (Roche, 20 mg/mL), 2.5 units of Taq polymerase (AmpliTaq Gold, Applied Biosystems), 250 µM of each dNTP (SIGMA) and finally 0.5 µM of each primers (Eurogentec). Four volumes of DNA extract were used for each amplification: 0.5 µL, 1.0 µL, 1.5 µL and 2.0 µL. Cycling conditions were the following: one activation step at 94°C for 10 min followed by 20 cycles of denaturation at 94°C for 30 s, annealing at 56°C for 30 s, elongation at 72°C for 45 s, and a final extension at 72°C for 5 min. The first PCR was used as a template (1 L of product diluted to 1/20 in sterile ultra pure water) for a second round of amplification using a single pair of primers. This second PCR reaction was performed in the same conditions as above except that 1.25 units of Taq polymerase (AmpliTaq Gold, Applied Biosystems) was used and cycling conditions were modified: one activation step at 94°C for 10 min followed by 40 cycles of denaturation at 94°C for 30 s, annealing at 58–62°C [depending on primer melting temperature] for 30 s, elongation at 72°C for 45 s, and a final extension at 72°C for 5 min. We repeated PCR sessions in order to obtain at least two independent amplifications for each sample (Table S3). Five µl of the PCR products were visualized on 2% agarose gels using ethidium bromide staining and UV light transillumination. Positive PCR products were purified using MinElute PCR Purification columns (Qiagen) and were quantified using the Quant-iT ™ dsDNA High-Sensitivity Assay Kit (Invitrogen). Subsequently equimolar amounts of each amplicon (up to 157 products for a run) were pooled to build each library. In order to differentiate the products after sequencing, all the primers used for PCR were tagged using Multiplex Identifiers (MIDs) designed by Roche. We used 454 technology (GS junior, Roche) to sequence amplicons: all procedures (i.e Library preparation, emPCR amplification, DNA bead enrichment and sequencing run) were carried out according to the Roche GS Junior protocols for amplicon sequencing. Authentic sequences were determined by analyzing reads obtained for each product (422 reads on average by amplicon) and by considering independent amplifications for each gene and sample. In particular, a high number of reads by amplicon was necessary to monitor artefactual substitutions and degradation patterns, well identified for aDNA [35]. Ancient canid sequences from this article have been deposited in the GenBank Data Library under accession numbers JQ971988 to JG972027.

### Modern DNA

Modern DNA work was performed after the aDNA analyses and in a distinct lab on 49 individuals. DNA samples from 43 present-day dogs belonging to 13 distinct modern breeds and 6 wolves come from the caniDNA Biobank developed at IGDR, CNRS-UMR6290, Rennes, France. The near entire *Mc1r* coding sequence was amplified using primers D and E (Newton et al., 2000); D 5′-GGTCATTGCTGAGCTGACAC-3′, and E, 5′-GAGATGCTGTCCAGTAGTCCC-3′. The same methods and conditions as described above applied to amplify a 942 bp *Mc1r* fragment except that only 40 cycles of PCR were performed. Positive PCR amplicons were cloned by using Topo TA Cloning for Sequencing kit (Invitrogen) (Table S5). At least 8 clones by-products were picked and amplified by PCR with the ready-to-use Mastermix (Eppendorf, Hamburg, Germany) using the following cycling conditions: a first break-up bacterial cell step at 94°C for 30 s followed by 40 cycles of denaturation at 94°C for 30 s, annealing at 55°C for 30 s, elongation at 72°C for 2 min, and a final extension at 72°C for 5 min. Products of the expected size were sequenced on both strands by Cogenics, Essex, United-Kingdom. These 49 sequences have been deposited in the GenBank Data Library nder accession numbers JX273571–JX273640.

### Mutation Analysis

454 data products were sorted using the public Galaxy web interface (http://www.galaxy.psu.edu/, [36]–[38]) through a dedicated pipeline. For each sample and gene, the reads obtained by 454-sequencing or sequences of present-day dogs and wolves that were cloned were aligned with SEAVIEW software (http://pbil.univ-lyon1.fr/software/seaview.html, [39]) and compared to reference sequences of the gene to detect SNPs (delivered by the Broad Institute -http://genome.cse.ucsc.edu/cgi-bin/hgGateway). As we studied nuclear DNA, individuals can be heterozygous or homozygous at a given locus. Thus we computed the frequency of each of the SNPs (number of reads or clones) for each individual and locus (Table S4, Table S5). The heterozygous or homozygous status was ascertained for ancient samples by considering independent amplicons and high coverage of reads.

## Supporting Information

Table S1 **Studied genes, alleles and SNP, implication of each allele in dog pigmentation and derived phenotypes.** Table S1 presents, for each studied genes (*Mc1r*, *CBD103*), the loci that were targeted. For each locus, allele dominance, allele status (wild or derived), SNP names, corresponding mutations are described as well as their respective implication in dog pigmentation. These data are derived from previous studies [22]–[23]. In a second table, indicative coat color phenotypes are given according to the SNP combinations of the two-targeted genes, which were previously described in Candille *et al* (2007) [23].
(DOC)

Table S2 **Archaeological site, location, radiometric and cultural dating and aDNA results for the 68 canids analyzed in this study.** Table S2 lists archaeological sites, locations, radiometric and cultural dating for the 68 canids analyzed in this study. For each sites the number of aDNA nuclear sequences obtained in this study and the number of samples tested are given for both *Mc1r* and *CBD103* genes.
(DOC)

Table S3 **Number of positive PCR compared to the number of attempts (Proportion/ratio of positive PCR vs number of attempts) and relative number of reads obtained with 454 sequencing for each sequencing product, for each gene, per sample.** Squares refer to individuals with a wolf-morphotype. *: For these samples, faint bands were obtained for other PCR products but no sequencing products were obtained after both 454 sequencing and cloning-sequencing. Table S3. lists samples for which positive PCR were obtained for either *Mc1r* or *CBD103*. The number of positive PCR is compared to the number of attempts and relative number of reads obtained with 454 sequencing for each sequencing product, for each gene, per sample is indicated. For six samples (CH989, CH770, CH740, CH1074, CH700, CH730), faint bands were obtained for other PCR products but no sequencing products were obtained after both 454 sequencing and cloning-sequencing. As they can correspond to artifacts, these PCR products were *a posteriori* not considered positive.
(DOCX)

Table S4 **Relative proportion (%) of reads per sequencing products that present** ***Mc1r*** **R301C,** ***Mc1r*** **R306ter or the** ***CBD103*** **ΔG23 mutations for 15 and 19 samples and allelic state deduce for these three loci.** Squares refer to individuals with a wolf-morphotype. Table S4 displays percentage of reads per sequencing products that present wild or derived state for the following three loci: *Mc1r* R301C, *Mc1r* R306ter or the *CBD103* ΔG23 mutations. Results are given for 15 and 19 samples for which respectively *Mc1r* and *CBD103* amplifications could have been replicated. For each sample the deduced allelic states for these three loci are given.
(DOCX)

Table S5 ***Mc1r*** **R301C and R306ter allelic states for 43 dogs belonging to 13 distinct modern breeds and 6 present day wolves.** Squares and dots refer to wolves and dogs, respectively. Brown: R301C mutation, homozygous state; Orange: R301C mutation, heterozygous state; Blue: R306ter mutation, homozygous state; light blue: R306ter mutation, heterozygous state. Table S5 lists *Mc1r* R301C and R306ter allelic states for 43 dogs belonging to 13 distinct modern breeds and 6 present day wolves. For each sample, we also provide the reference in the CaniDNA biobank (IGDR, CNRS-UMR6290, Rennes, France) and the breed (for dog samples) or subspecies (for wolf samples). For both locus and for each sample, we indicate the number of clones with either the R301C or the R306ter mutation. R301C is found in 11 dogs belonging to Siberian husky or Alaskan malamute breeds at heterozygous (orange) or homozygous (brown) state. The R306ter premature stop codon, in the dog melanocortin receptor 1 (*MC1R*) gene, is present in 12 dogs belonging to 10 breeds in the heterozygous (light blue) or in the homozygous state (blue) in dogs in dogs with red or yellow coat color [22]–[29].
(DOCX)
