# Supplementary material for: Evidence of Coat Color Variation Sheds New Light on Ancient Canids
Source: PLoS One. 2013 Oct 2;8(10):e75110. doi: 10.1371/journal.pone.0075110 (PMC3788791; doi:10.1371/journal.pone.0075110)
Supplement: Table S1 — Studied genes, alleles and SNP, implication of each allele in dog pigmentation and derived phenotypes. Table S1 presents, for each studied genes (Mc1r, CBD103), the loci that were targeted. For each locus, allele dominance, allele status (wild or derived), SNP names, corresponding mutations are described as well as their respective implication in dog pigmentation. These data are derived from previous studies [22]–[23]. In a second table, indicative coat color phenotypes are given according to the SNP combinations of the two-targeted genes, which were previously described in Candille et al (2007) [23]. (DOC) [file pone.0075110.s001.doc]

**Table S1:** Studied genes, alleles and SNP, implication of each allele in dog pigmentation and derived phenotypes *(1)*.

| **Genes** | **Allele dominance** | **SNP** | **Allele** | **Mutation** | **Implication in dog pigmentation** |
| --- | --- | --- | --- | --- | --- |
| ***Mc1r*** | E > e | R306ter *(2)* | E (wild) | C | Eumelanin can be produced |
|  |  |  | e | T | Only phaeomelanin can be produced |
| ***CBD103*** | K^B^ > K^y^ | ΔG23 *(1)* | K^y^ (wild) | GGG | Expression phaeomelanin is possible |
|  |  |  | K^B^ | --- | Eumelanin pigmentation only (black) |

**Derived phenotypes:**

| **Coat color phenotypes** | **Alleles** *Mc1r* / *CDB103* | **Mutations** *Mc1r* / *CDB103* |
| --- | --- | --- |
| Wild yellow | E (wild) / K^y^ (wild) | C / GGG |
| Recessive yellow | e / K^y^ or K^B^ | T / GGG or --- |
| Dominant black | E (wild) / K^B^ | C / --- |

**References**:

1. Candille SI, *et al.* (2007) A beta-defensin mutation causes black coat color in domestic dogs. *Science* **318**, 1418.
2. Newton JM, *et al.* (2000) Melanocortin 1 receptor variation in the domestic dog. *Mammalian Genome* **11**, 24.
