# Supplementary material for: Evidence of Coat Color Variation Sheds New Light on Ancient Canids
Source: PLoS One. 2013 Oct 2;8(10):e75110. doi: 10.1371/journal.pone.0075110 (PMC3788791; doi:10.1371/journal.pone.0075110)
Supplement: Table S2 — Archaeological site, location, radiometric and cultural dating and aDNA results for the 68 canids analyzed in this study. Table S2 lists archaeological sites, locations, radiometric and cultural dating for the 68 canids analyzed in this study. For each sites the number of aDNA nuclear sequences obtained in this study and the number of samples tested are given for both Mc1r and CBD103 genes. (DOC) [file pone.0075110.s002.doc]

**Table S2**: Archaeological site, location, radiometric and cultural dating and aDNA results for the 68 canids analyzed in this study.

| **Archeological Site** | **Date obtained directly on dog bones (cal BP)** | **Date derived from other remains (BP)** | **Chronological periods derived from cultural attributions** | **Cultural period** | **Nuclear sequences obtained */* Individual tested** | |
| --- | --- | --- | --- | --- | --- | --- |
|  |  |  |  |  | *Mc1r* | *CBD103* |
| *Sagzabad* |  | [3150-2950] |  | **IRON AGE Iran** | 0/1 | 0/1 |
|  |  |  |  |  |  |  |
| *Bury* | [4451-4247] |  |  | **BRONZE AGE France** | 2/2 | 2/2 |
|  |  |  |  |  |  |  |
| *Luka Vrubiletskaia* |  |  | 4950-4650 | **CHALCOLITHIC Cucuteni culture** | 0/1 | 1/1 |
| *Soloncheny* |  |  | 4950-4650 |  | 0/1 | 1/1 |
|  |  |  |  |  |  |  |
| *Ulug Depe* |  | [5450-3950] |  | **BRONZE AGE Turkmenistan** | 1/1 | 1/1 |
| *Altyn Depe* |  |  | 5700-5000 |  | 0/1 | 0/1 |
|  |  |  |  |  |  |  |
| *Saint Paul Trois Château* |  |  | 5900-5600 | **NEOLITHIC Chasséen Culture Cortaillod Culture Paris Basin Jura Rhône Valley** | 0/2 | 0/2 |
| *Twann* |  |  | 6150-5400 |  | 1/5 | 1/5 |
| *Chalain-Clairvaux* |  |  | 6500-5500 |  | 0/2 | 0/2 |
| *Noyen-sur-Seine* |  |  | 6500-5900 |  | 0/2 | 0/2 |
| *Châtenay-sur-Seine* |  |  | 6500-5900 |  | 0/1 | 0/1 |
| *Bercy* |  | [6311-5875] |  |  | 0/3 | 0/3 |
|  |  |  |  |  |  |  |
| *Hârșova* |  |  | 6500-5900 | **CHALCOLITHIC Boian and Gumelnita culture Romania** | 2/4 | 2/4 |
| *Bordușani* | [6798-6674] |  | 6798-5900 |  | 0/7 | 1/7 |
| *Silistea* |  |  | 6950-6450 |  | 0/1 | 0/1 |
|  |  |  |  |  |  |  |
| *Herxheim* |  | [6995-6145] | 7500-6900 | **NEOLITHIC LBK Culture Germany** | 1/3 | 1/3 |
|  |  |  |  |  |  |  |
| *Isacea* |  | [7440-7318] |  | **NEOLITHIC Romania** | 1/2 | 1/2 |
|  |  |  |  |  |  |  |
| *Tepe Sang-e-Caxmaq* | [8067-7794] |  |  | **EARLY NEOLITHIC Iran** | 0/7 | 0/7 |
|  |  |  |  |  |  |  |
| *Narva I, II, III* | [7161-5583] |  |  | **MESOLITHIC Narva Culture Estonia** | 0/4 | 0/4 |
|  |  |  |  |  |  |  |
| *Téviec* |  |  | 7650-7450 | **LATE MESOLITHIC French Britany** | 0/2 | 0/2 |
|  |  |  |  |  |  |  |
| *Ust'belaya* |  | [10233-9909] |  | **MESOLITHIC Siberia** | 3/3 | 3/3 |
| *Pad'Kalashnikova* |  |  | 10500-9000 |  | 2/2 | 2/2 |
|  |  |  |  |  |  |  |
| *Ostrovul Corbului* |  | [9522-8320] |  | **EARLY and LATE MESOLITHIC**  **Iron Gates** | 0/1 | 0/1 |
| *Icoana* |  | [10345-8050] |  | **EARLY and LATE MESOLITHIC Iron Gates EARLY MESOLITHIC Iron Gates** | 2/4 | 2/4 |
| *Cuina Turcului* |  | [15246-11212] |  |  | 0/1 | 0/1 |
|  |  |  |  |  |  |  |
| *Pont d’Ambon* | [12952-12451] |  |  | **EPI-PALEOLITHIC Azilian Culture Kebarian** | 0/1 | 0/1 |
| *Hayonim* |  |  | 15500-11000 |  | 0/1 | 0/1 |
| *Torgashinskaya cave* |  |  | 15000-12000 |  | 1/1 | 0/1 |
|  |  |  |  |  | **15/68** | **19/68** |

For each gene, we indicate the number of samples tested and the number of authenticated sequences obtained.
