# Supplementary material for: Evidence of Coat Color Variation Sheds New Light on Ancient Canids
Source: PLoS One. 2013 Oct 2;8(10):e75110. doi: 10.1371/journal.pone.0075110 (PMC3788791; doi:10.1371/journal.pone.0075110)
Supplement: Table S3 — Number of positive PCR compared to the number of attempts (Proportion/ratio of positive PCR vs number of attempts) and relative number of reads obtained with 454 sequencing for each sequencing product, for each gene, per sample. Squares refer to individuals with a wolf-morphotype. *: For these samples, faint bands were obtained for other PCR products but no sequencing products were obtained after both 454 sequencing and cloning-sequencing. Table S3. lists samples for which positive PCR were obtained for either Mc1r or CBD103. The number of positive PCR is compared to the number of attempts and relative number of reads obtained with 454 sequencing for each sequencing product, for each gene, per sample is indicated. For six samples (CH989, CH770, CH740, CH1074, CH700, CH730), faint bands were obtained for other PCR products but no sequencing products were obtained after both 454 sequencing and cloning-sequencing. As they can correspond to artifacts, these PCR products were a posteriori not considered positive. (DOCX) [file pone.0075110.s003.docx]

**Table S3:** Number of positive PCR compared to the number of attempts (Proportion / ratio of positive PCR vs number of attempts) and relative number of reads obtained with 454 sequencing for each sequencing product, for each gene, per sample. *In italic*: individuals with a wolf-morphotype (CH1075 and CH1244) *: For these samples, faint bands were obtained for other PCR products but no sequencing products were obtained after both 454 sequencing and cloning-sequencing.

|  |  |  | **MC1R (79bp)** | | **CBD103 (83bp)** | |
| --- | --- | --- | --- | --- | --- | --- |
| **Sample reference** | **Country** | **Site** | **Number of positive PCR / number of attempts** | **Number of 454 sequencing reads by PCR product** | **Number of positive PCR / number of attempts** | **Number of 454 sequencing reads by PCR product** |
| CH734 | France | Bury | 3/6 | 919 / 721 / 667 | 2/7 | 451 / 153 |
| CH735 |  |  | 2/4 | 404 / 340 | 2/5 | 543 / 505 |
| CH716 | Ukraine | Luka Vrubiletskaia | 0/4 | - | 2/4 | 216 / 123 |
| CH717 | Moldavia | Soloncheny | 0/4 | - | 2/7 | 82 / 200 |
| *CH1075* | Turkmenistan | Ulug Depe | 2/5 | 134 / 998 | 4/10 | 87 / 135 / 204 / 764 |
| CH1076 |  |  | 0/4 | - | 2/7 | 233 / 378 |
| CH756 | France | Saint Paul Trois Chateaux | 0/7 | - | 3/8 | 486 / 523 / 292 |
| CH1047 | Switzerland | Twann | 2/11 | 685 / 528 | 0/12 |  |
| CH773 | Romania | Bordușani | 0/4 | - | 2/9 | 81 / 47 |
| CH989* |  |  | 0/6 | - | 1/13 | 902 |
| CH768 |  | Hârșova | 0/4 | - | 2/5 | 209 / 239 |
| CH770* |  |  | 2/15 | 1150 / 7 | 0/5 | - |
| CH771 |  |  | 4/12 | 414 / 638 / 516 / 648 | 2/6 | 244 / 261 |
| CH1042 | Germany | Herxheim | 3/3 | 410 / 460 / 534 | 2/7 | 293 / 207 |
| CH767 | Romania | Isaccea | 4/11 | 512 / 509 / 84 / 1518 | 2/12 | 40 / 356 |
| CH740* | Iran | Tepe Sang-i-Caxmaq | 0/5 | - | 1/11 | 42 |
| CH1074* |  |  | 0/6 | - | 1/7 | 648 |
| CH700* | France | Téviec | 0/9 | - | 0/12 |  |
| CH708 | Russia | Pad'Kalashnikova | 4/4 | 2213 / 228 / 1138 / 1110 | 2/3 | 184 / 168 |
| CH709 |  |  | 3/4 | 1000 / 287 / 353 | 3/4 | 386 / 322 / 744 |
| CH710 |  | Ust'Belaya | 0/6 | - | 2/5 | 141 / 33 |
| CH711 |  |  | 4/4 | 379 / 591 / 388 / 806 | 3/4 | 86 / 189 / 76 |
| CH712 |  |  | 3/6 | 1537 / 317 / 12294 | 2/5 | 500 / 177 |
| CH1119 | Romania | Icoana | 0/6 | - | 2/8 | 82 / 107 |
| CH1120 |  |  | 2/14 | 552 / 180 | 2/12 | 253 / 295 |
| CH1122 |  |  | 2/13 | 1094 / 106 | 0/6 | - |
| CH730* | France | Pont d'Ambon | 0/4 | - | 1/8 | 264 |
| *CH1244* | Russia | Torgashinskaya cave | 2/13 | 479 / 984 | 1/11 | 116 |
