# Supplementary material for: Evidence of Coat Color Variation Sheds New Light on Ancient Canids
Source: PLoS One. 2013 Oct 2;8(10):e75110. doi: 10.1371/journal.pone.0075110 (PMC3788791; doi:10.1371/journal.pone.0075110)
Supplement: Table S4 — Relative proportion (%) of reads per sequencing products that present Mc1r R301C, Mc1r R306ter or the CBD103 ΔG23 mutations for 15 and 19 samples and allelic state deduce for these three loci. Squares refer to individuals with a wolf-morphotype. Table S4 displays percentage of reads per sequencing products that present wild or derived state for the following three loci: Mc1r R301C, Mc1r R306ter or the CBD103 ΔG23 mutations. Results are given for 15 and 19 samples for which respectively Mc1r and CBD103 amplifications could have been replicated. For each sample the deduced allelic states for these three loci are given. (DOCX) [file pone.0075110.s004.docx]

**Table S4**: Relative proportion (%) of reads per sequencing products that present *Mc1r* R301C, *Mc1r* R306ter or the *CBD103* ΔG23 mutations for 15 and 19 samples and allelic state deduce for these three loci. *In italic*: individuals with a wolf-morphotype (CH1075 and CH1244).

| **Sample reference** | **Country** | **Site** | ***Mc1r* R301C (79bp)** | | | ***Mc1r* R306ter (79bp)** | | | ***CBD103* ΔG23 (83bp)** | | |
| --- | --- | --- | --- | --- | --- | --- | --- | --- | --- | --- | --- |
|  |  |  | % of C / replicate | % of T / replicate | Allelic state | % of C / replicate | % of T / replicate | Allelic state | % of GGG / replicate | % of --- / replicate | Allelic state |
| CH734 | France | Bury | 100 100 100 | 0 0 0 | C / C | 100 100 100 | 0 0 0 | C / C | 0 0 | 100 100 | --- / --- |
|  |  |  |  |  |  |  |  |  |  |  |  |
|  |  |  |  |  |  |  |  |  |  |  |  |
| CH735 |  |  | 100 100 | 0 0 | C / C | 100 100 | 0 0 | C / C | 100 100 | 0 0 | GGG / GGG |
| CH716 | Ukraine | Luka Vrubiletskaia | - | - | - | - | - | - | 100 100 | 0 0 | GGG / GGG |
| CH717 | Moldavia | Soloncheny | - | - | - | - | - | - | 100 100 | 0 0 | GGG / GGG |
| *CH1075* | Turkmenistan | Ulug Depe | 100 100 | 0 0 | C / C | 100 100 | 0 0 | C / C | 0 100 40 46 | 100 0 60 54 | GGG / --- |
| CH1076 |  |  | - | - | - | - | - | - | 100 100 | 0 0 | GGG / GGG |
| CH756 | France | Saint Paul Trois Chateaux | - | - | - | - | - | - | 100 100 100 | 0 0 0 | GGG / GGG |
| CH1047 | Switzerland | Twann | 100 100 | 0 0 | C / C | 100 100 | 0 0 | C / C | - | - | - |
| CH773 | Romania | Bordușani | - | - | - | - | - | - | 100 100 | 0 0 | GGG / GGG |
| CH768 |  | Hârșova | - | - | - | - | - | - | 100 100 | 0 0 | GGG / GGG |
| CH770 |  |  | 0 0 | 100 100 | T / T | 100 100 | 0 0 | C / C | - | - | - |
| CH771 |  |  | 0 100 0 75 | 100 0 100 25 | C / T | 100 100 100 100 | 0 0 0 0 | C / C | 0 0 | 100 100 | --- / --- |
| CH1042 | Germany | Herxheim | 100 100 100 | 0 0 0 | C / C | 100 100 100 | 0 0 0 | C / C | 100 100 | 0 0 | GGG / GGG |
| CH767 | Romania | Isaccea | 0 0 0 0 | 100 100 100 100 | T / T | 100 100 100 100 | 0 0 0 0 | C / C | 0 44 | 100 56 | GGG / --- |
| CH708 | Russia | Pad'Kalashnikova | 58 28 67 46 | 42 72 33 54 | C / T | 100 100 100 100 | 0 0 0 0 | C / C | 100 100 | 0 0 | GGG / GGG |
| CH709 |  |  | 68 57 31 | 32 43 69 | C / T | 100 100 100 | 0 0 0 | C / C | 100 100 100 | 0 0 0 | GGG / GGG |
| CH710 |  | Ust'Belaya | - | - | - | - | - | - | 100 100 | 0 0 | GGG / GGG |
| CH711 |  |  | 55 36 50 80 | 45 64 50 20 | C / T | 100 100 100 100 | 0 0 0 0 | C / C | 100 100 100 | 0 0 0 | GGG / GGG |
| CH712 |  |  | 0 0 1 | 100 100 99 | T / T | 100 100 100 | 0 0 0 | C / C | 100 100 | 0 0 | GGG / GGG |
| CH1119 | Romania | Icoana | - | - | - | - | - | - | 100 100 | 0 0 | GGG / GGG |
| CH1120 |  |  | 0 0 | 100 100 | T / T | 100 100 | 0 0 | C / C | 0 0 | 100 100 | --- / --- |
|  |  |  |  |  |  |  |  |  |  |  |  |
| CH1119 |  |  | 0 0 | 100 100 | T / T | 100 100 | 0 0 | C / C | - | - | - |
| *CH1244* | Russia | Torgashinskaya cave | 100 100 | 0 0 | C / C | 100 100 | 0 0 | C / C | - | - | - |
