# Supplementary material for: Evidence of Coat Color Variation Sheds New Light on Ancient Canids
Source: PLoS One. 2013 Oct 2;8(10):e75110. doi: 10.1371/journal.pone.0075110 (PMC3788791; doi:10.1371/journal.pone.0075110)
Supplement: Table S5 — Mc1r R301C and R306ter allelic states for 43 dogs belonging to 13 distinct modern breeds and 6 present day wolves. Squares and dots refer to wolves and dogs, respectively. Brown: R301C mutation, homozygous state; Orange: R301C mutation, heterozygous state; Blue: R306ter mutation, homozygous state; light blue: R306ter mutation, heterozygous state. Table S5 lists Mc1r R301C and R306ter allelic states for 43 dogs belonging to 13 distinct modern breeds and 6 present day wolves. For each sample, we also provide the reference in the CaniDNA biobank (IGDR, CNRS-UMR6290, Rennes, France) and the breed (for dog samples) or subspecies (for wolf samples). For both locus and for each sample, we indicate the number of clones with either the R301C or the R306ter mutation. R301C is found in 11 dogs belonging to Siberian husky or Alaskan malamute breeds at heterozygous (orange) or homozygous (brown) state. The R306ter premature stop codon, in the dog melanocortin receptor 1 (MC1R) gene, is present in 12 dogs belonging to 10 breeds in the heterozygous (light blue) or in the homozygous state (blue) in dogs in dogs with red or yellow coat color [22]–[29]. (DOCX) [file pone.0075110.s005.docx]

**Table S5**: *Mc1r* R301C and R306ter allelic states for 43 dogs belonging to 13 distinct modern breeds and 6 present day wolves. In bold: presence of the R301C or R306ter mutation either at homozygous or heterozygous state.

The R306ter premature stop codon, in the dog melanocortin receptor 1 (MC1R) gene, is present in the homozygous state in dogs in dogs with red or yellow coat color *(1, 2)*.

| **Species** | **Sample reference** | **Breed or subspecies** | ***MC1R* R301C (this study)** | | ***MC1R* R306ter (described in Newton 2000) *(1*,*2)*** | | |
| --- | --- | --- | --- | --- | --- | --- | --- |
|  |  |  | ***Number of clones with the R301C mutation (C -> T)*** | ***Allelic state*** | ***Number of clones with the R306ter mutation (C -> T)*** | | ***Allelic state*** |
| *Canis lupus familiaris* | Atout Coeur | Siberian Husky | 5/12 | **T / C** | 0/12 | C / C | |
|  | Dayak |  | 0/12 | C / C | 0/12 | C / C | |
|  | Farell |  | 6/12 | **T / C** | 0/12 | C / C | |
|  | Shelly |  | 4/12 | **T / C** | 0/12 | C / C | |
|  | Ambre |  | 5/8 | **T / C** | 0/8 | C / C | |
|  | Fraie |  | 10/10 | **T / T** | 0/10 | C / C | |
|  | Damyka | Alaskan malamute | 11/11 | **T / T** | 0/11 | C / C | |
|  | Phantom |  | 12/12 | **T / T** | 0/12 | C / C | |
|  | Unaaq |  | 10/10 | **T / T** | 0/10 | C / C | |
|  | Bandit |  | 12/12 | **T / T** | 0/12 | C / C | |
|  | Heilani |  | 4/12 | **T / C** | 5/12 | **T / C** | |
|  | Jenny |  | 12/12 | **T / T** | 0/12 | C / C | |
|  | 7728 | Sharpei | 0/7 | C / C | 0/7 | C / C | |
|  | 7826 |  | 0/8 | C / C | 3/8 | **T / C** | |
|  | 8238 |  | 0/8 | C / C | 1/8 | **T / C** | |
|  | 7220 | Chowchow | 0/8 | C / C | 8/8 | **T / T** | |
|  | 7663 |  | 0/8 | C / C | 0/8 | C / C | |
|  | 8248 |  | 0/8 | C / C | 8/8 | **T / T** | |
|  | 6347 | Akita | 0/8 | C / C | 0/8 | C / C | |
|  | 6430 |  | 0/7 | C / C | 6/7 | **T / C** | |
|  | 6495 |  | 0/8 | C / C | 0/8 | C / C | |
|  | 7622 |  | 0/8 | C / C | 4/8 | **T / C** | |
|  | 3298 | Eurasier | 0/8 | C / C | 0/8 | C / C | |
|  | 5272 |  | 0/8 | C / C | 0/8 | C / C | |
|  | 5985 |  | 0/8 | C / C | 5/8 | **T / C** | |
|  | 2960 | Greyhound | 0/8 | C / C | 0/8 | C / C | |
|  | 3159 |  | 0/7 | C / C | 0/7 | C / C | |
|  | 3277 |  | 0/8 | C / C | 0/8 | C / C | |
|  | 1685 | Saluki | 0/8 | C / C | 8/8 | **T / T** | |
|  | 1686 |  | 0/8 | C / C | 0/8 | C / C | |
|  | 1687 |  | 0/7 | C / C | 0/7 | C / C | |
|  | 4675 | Dogue du tibet | 0/7 | C / C | 0/7 | C / C | |
|  | 4676 |  | 0/8 | C / C | 0/8 | C / C | |
|  | 8210 |  | 0/8 | C / C | 0/8 | C / C | |
|  | 6637 | Boxer | 0/8 | C / C | 0/8 | C / C | |
|  | 6801 |  | 0/8 | C / C | 0/8 | C / C | |
|  | 6805 |  | 0/8 | C / C | 0/8 | C / C | |
|  | 5212 | Samoyède | 0/8 | C / C | 8/8 | **T / T** | |
|  | SAM |  | 0/7 | C / C | 7/7 | **T / T** | |
|  | 5128 | Chihuahua | 0/7 | C / C | 0/7 | C / C | |
|  | 6454 |  | 0/7 | C / C | 0/7 | C / C | |
|  | 7423 | Beagle | 0/7 | C / C | 4/7 | **T / C** | |
|  | 7947 |  | 0/8 | C / C | 0/8 | C / C | |
| *Canis lupus* | 6812 | Arctic wolf | 0/8 | C / C | 0/8 | C / C | |
|  | 7056 |  | 0/8 | C / C | 0/8 | C / C | |
|  | 6813 | Grey wolf | 0/12 | C / C | 0/12 | C / C | |
|  | 7036 |  | 0/11 | C / C | 0/11 | C / C | |
|  | 7180 |  | 0/12 | C / C | 0/12 | C / C | |
|  | 7269 | Undetermined | 0/12 | C / C | 0/12 | C / C | |

**References:**

1. Newton JM, *et al.* (2000) Melanocortin 1 receptor variation in the domestic dog. *Mammalian Genome* **11**, 24.
2. Everts RE, Rothuizen J, van Oost BA (2000) Identification of a premature stop codon in the melanocyte-stimulating hormone receptor gene (*MC1R*) in Labrador and Golden retrievers with yellow coat colour. *Animal Genetics* **31**, 194.
